# Functional binding of E-selectin to its ligands is enhanced by structural features beyond its lectin domain

**DOI:** 10.1074/jbc.RA119.010910

**Published:** 2020-01-16

**Authors:** Fajr A. Aleisa, Kosuke Sakashita, Jae Man Lee, Dina B. AbuSamra, Bader Al Alwan, Shuho Nozue, Muhammad Tehseen, Samir M. Hamdan, Satoshi Habuchi, Takahiro Kusakabe, Jasmeen S. Merzaban

**Affiliations:** ‡ Division of Biological and Environmental Sciences and Engineering (BESE), King Abdullah University of Science and Technology (KAUST), Thuwal, Saudi Arabia, 23955-6900; § Laboratory of Insect Genome Science, Kyushu University Graduate School of Bioresource and Bioenvironmental Sciences, Hakozaki 6-10-1, Higashi-ku, Fukuoka 812-8581, Japan

**Keywords:** cell migration, cell adhesion, glycoprotein structure, kinetics, glycobiology, carbohydrate-binding protein, dimerization, binding kinetics, complement regulatory repeats, E-selectin, short consensus repeats, silkworm expression, adhesion, glycoprotein, conformational extension, homing, silkworm expression

## Abstract

Selectins are key to mediating interactions involved in cellular adhesion and migration, underlying processes such as immune responses, metastasis, and transplantation. Selectins are composed of a lectin domain, an epidermal growth factor (EGF)-like domain, multiple short consensus repeats (SCRs), a transmembrane domain, and a cytoplasmic tail. It is well-established that the lectin and EGF domains are required to mediate interactions with ligands; however, the contributions of the other domains in mediating these interactions remain obscure. Using various E-selectin constructs produced in a newly developed silkworm-based expression system and several assays performed under both static and physiological flow conditions, including flow cytometry, glycan array analysis, surface plasmon resonance, and cell-rolling assays, we show here that a reduction in the number of SCR domains is correlated with a decline in functional E-selectin binding to hematopoietic cell E- and/or L-selectin ligand (HCELL) and P-selectin glycoprotein ligand-1 (PSGL-1). Moreover, the binding was significantly improved through E-selectin dimerization and by a substitution (A28H) that mimics an extended conformation of the lectin and EGF domains. Analyses of the association and dissociation rates indicated that the SCR domains, conformational extension, and dimerization collectively contribute to the association rate of E-selectin–ligand binding, whereas just the lectin and EGF domains contribute to the dissociation rate. These findings provide the first evidence of the critical role of the association rate in functional E-selectin–ligand interactions, and they highlight that the SCR domains have an important role that goes beyond the structural extension of the lectin and EGF domains.

## Introduction

The multistep paradigm of cell migration outlines a sequence of events that leads cells (*e.g.* stem cells, leukocytes, or circulating tumor cells) out of the circulating blood and into a target organ/tissue. This sequence of events, called homing, is initiated by the interaction of selectins (specifically, E-, P-, and L-selectins) with their ligands (1–3). Although each step in this process is important, the interactions mediating the first step of homing are crucial to slowing the circulating cells interacting with the endothelium to velocities that are lower than the local flow rate of cells in the blood. The most effective contributors to this critical step are the selectins.

E-selectin is constitutively expressed on bone marrow endothelium, where it recruits circulating hematopoietic stem/progenitor cells (HSPC)^2^ from the blood and is also important for the maintenance of HSPC within the niche (4, 5). E-selectin shows an affinity toward a prototypic sialylated and fucosylated structure known as sialyl-Lewis X (sLe^x^) (NeuAcα2–3Gal1-4(Fucα1–3)GlcNAc1-R) (2, 6), which is expressed on glycoproteins and glycolipids (7–9). Studies suggest that the primary interaction with these ligands occurs through the carbohydrate-binding lectin domain (10), but less is known about the influence of the remaining structural components of the selectin molecule and how they may contribute to the functional binding activity.

The selectins are structurally composed of five distinct domains: an N-terminal extracellular C-type lectin-like domain, followed by an endothelial growth factor (EGF)–like domain, a defined number of short consensus repeats (SCRs) with 60 amino acids per motif, a transmembrane domain, and a C-terminal cytoplasmic tail that is likely involved in signal transduction regulation (11–15). Although the three selectins share similar structures, they differ in both binding specificity toward their ligands and the number of SCR domains. Although the lectin and EGF domains have a high degree of homology, that of their SCR domains is significantly lower (16). Several studies elucidated the role of the lectin and EGF domains in the interaction of selectins with their ligands (17–19), but the role of the SCR domains in this interaction remains elusive. It is speculated that these domains act as structural spacers that extend the lectin-binding domain beyond the crowded glycocalyx of the cell surface (11). However, studies where the SCR domains were deleted suggest that they may play a role in E-selectin–ligand interactions (20–22). Nonetheless, these studies do not provide information about the functional role of SCRs in the binding of E-selectin, especially under physiological flow conditions.

Studies using P-selectin suggest that it adopts a bent (lower affinity) conformation in the absence of its sulfated ligand but in its presence shifts toward an extended (higher affinity) conformation (22). Moreover, P-selectin is found as both a monomer and a dimer/oligomer on activated platelets. This heterogeneity in P-selectin structure is suggested to contribute to tethering through the low-affinity monomeric structures, whereas, at later stages, the dimerization of extended structures may facilitate stronger binding to stabilize interactions during the rolling process (23). It is unclear if and how dimerization influences the E-selectin binding, or whether the formation of an extended conformer that opens the angle between the lectin and EGF domains influences its binding in a manner analogous to that observed in P-selectin.

**Figure 1.**
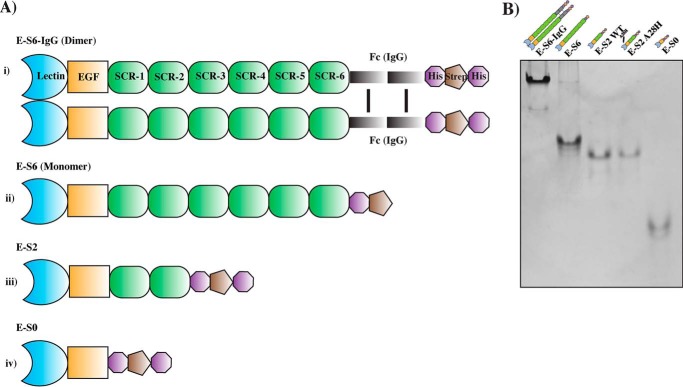
**Expression and purification of functional E-selectin proteins.** *A*, schematic representation of E-selectin constructs. *i*, E-S6-IgG consisting of the structural domains of a commonly used E-selectin-IgG/Fc (dimer) chimera recombinant protein. *ii*, monomeric version of full-length E-selectin (E-S6). *iii* and *iv*, truncated E-selectin formed by domain deletion of either the last four SCRs, producing constructs with only the first two SCRs (E-S2), or deletion of all of the SCRs, yielding a minimal construct possessing only the main domains for binding (E-S0). Several tags were included at the C terminus of each construct, such as a histidine tag (double His_6_ or a single His_8_) and a Strep-tag, to facilitate the purification and subsequent immobilization of E-selectins (note that an Avi tag located after the EGF domain in E-S6-IgG, E-S2, and E-S0 and a TEV tag included before His_8_ in E-S6 are omitted from this diagram as they were not addressed in this study). *B*, analytical native PAGE of the recombinant E-selectin proteins. E-S6-IgG, E-S6, E-S2, E-S2-A28H, and E-S0 were diluted in 1× Native PAGE buffer, loaded on a 10% TBE gel and run in 1× Tris-glycine buffer. The majority of molecules in each recombinant protein sample appear as a single species, with a minor amount of oligomerization in case of E-S6; the majority of molecules in E-S6-IgG appear as a dimer through the C terminus Fc region, with some slightly monomeric versions appearing as a faint band of lower molecular weight.

In this study, we generated five recombinant E-selectin proteins (see Fig. 1*A*) secreted by a novel silkworm expression system that included dimeric and monomeric forms harboring varying numbers of SCR domains as well as an enforced extended conformer. These E-selectin proteins allowed us to demonstrate the influence of the various structural domains and conformational extension on the overall ability of E-selectin to bind its ligands. By employing a comprehensive set of assays under static and physiological flow conditions, we showed that SCR domains, the conformational extension of the lectin and EGF domains, and protein dimerization contribute to the efficient binding of E-selectin to its glycosylated ligands. Moreover, these three structural elements collectively contribute to the association rate, whereas the dissociation rate was mainly controlled by the lectin and EGF domains. These findings correlate, for the first time, a very important physiological role for the association rate in the functional binding of the selectin to its ligands.

## Results

### Active E-selectin proteins are expressed and purified from silkworm

To overcome the low level of eukaryotic proteins expressed by the widely used NS0 cells, we chose, for the first time, to use the silkworm expression system for E-selectin protein production (24). This system provides several advantages: it can synthesize and process signal peptides of secreted proteins, it has the machinery to produce proper folding, it can incorporate posttranslational modifications such as glycosylation and phosphorylation, and it is cost-effective (25). It is important to note that the type of *N*-glycosylation events that take place in insect cells are mainly pauci-mannose whereas in mammalian cells, they are more complex (26, 27) (Fig. S1). The differences in glycosylation patterns that result can affect the activity, stability, and solubility of the protein (28). Although a significant amount of the molecular weight of E-selectin is because of *N*-linked glycosylation, the protein-binding activity is not affected by the removal of *N*-glycans (29).

A Western blot analysis comparing the binding of two common E-selectin ligands, CD44/HCELL (hematopoietic cell E- and/or L-selectin ligand) and PSGL-1 (P-selectin glycoprotein ligand-1) (30–33), to dimeric E-selectin produced in silkworm showed similar binding patterns to that produced in a mammalian system (Fig. S2). This observation highlights the suitability of silkworm as an alternative system for expressing E-selectin.

To address the role of SCR domains and protein dimerization in the binding activity of E-selectin, various E-selectin constructs with varying numbers of SCR domains were generated (Fig. 1*A*): i) E-S6-IgG, which is a full-length dimeric protein that consists of a lectin domain followed by an EGF-like domain; six SCR domains; and an Fc region of IgG that aids in the dimerization of the molecule; ii) E-S6, which is a monomeric version of E-S6-IgG lacking the Fc region; iii) E-S2, which is a truncated protein with only the first two SCR domains; and iv) E-S0, which lacks any SCR domains. These protein constructs were successfully purified and did not appear to aggregate (Fig. 1*B*). On the other hand, the dimeric E-selectin, E-S6-IgG, was primarily dimerized (as facilitated by the Fc region) with a small amount showing up as a monomer (faint band in Fig. 1*B*). These observations suggest an absence of aggregation among the E-selectin proteins purified from the silkworm system.

### The number of SCR domains, structural extension, and dimerization of E-selectin influence binding to its ligands in both static and flow-based assays

**Figure 2.**
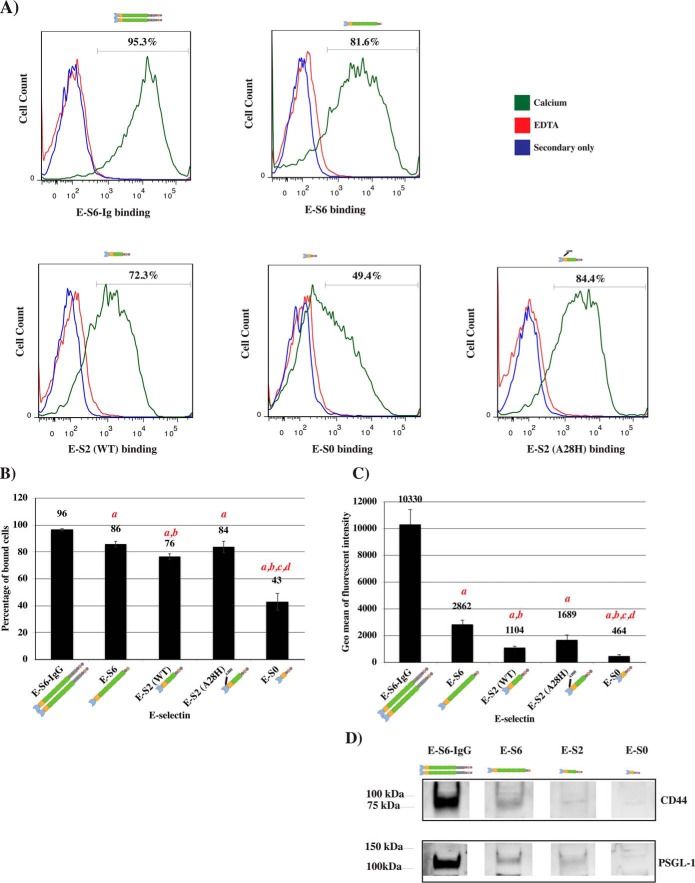
**Flow cytometric and Western blotting analyses comparing the binding functionalities of the various forms of E-selectin proteins to KG1a cells.** *A*, E-S6-IgG, E-S6, E-S2, E-S2-A28H, and E-S0 were tested for their ability to bind to ligands on KG1a cells. Mouse monoclonal anti-Strep antibody (followed by a fluorescently labeled antibody against anti-Strep) was used to detect the E-selectin protein bound to ligands on the surface of KG1a cells in the presence of calcium (*green* histogram) or EDTA (*red* histogram). Samples stained with only the secondary antibody are included as a control to determine secondary antibody specificity toward mouse monoclonal anti-Strep antibody (*blue* histogram). *B* and *C*, the percentages of KG1a cells bound to each of the E-selectin proteins and the geometric means of the fluorescence signals were determined from *n* = *3* independent experiments (*n* = *3*; *a*, indicates significance compared with E-S6-IgG; *b*, indicates significance compared with E-S6; *c*, indicates significance compared with E-S2; and *d*, indicates significance compared with E-S2-A28H, *p* ≤ 0.05) and the means are depicted as mean ± S.E.M. in (*B*) and (*C*), respectively. *D*, Western blot analysis of E-selectin protein binding to immunoprecipitated PSGL-1 and CD44. KG1a lysates were prepared, and PSGL-1 and CD44 were immunoprecipitated and subjected to the Western blot analysis. The resulting blots (*upper panel*, CD44; *lower panel*, PSGL-1) were stained with 1 μg/ml of E-S6-IgG, E-S6, E-S2, or E-S0, as indicated in the figure, in the presence of calcium. Anti-strep mAb was used against bound E-selectin for subsequent chemiluminescence detection using HRP-conjugated anti-mouse IgG. Blots stained in the presence of EDTA to confirm binding specificity showed no binding activity (Fig. S4).

We used flow cytometry to confirm the binding functionality of our recombinant E-selectin proteins on known E-selectin ligands under native conditions. In these experiments, KG1a cells were used to represent HSPC-like (CD34^+^) cells, which are able to bind E-selectin (30, 31). The E-selectin expressed and purified from the silkworm was incubated with KG1a cells to allow them to interact with the different ligands expressed on these cells. The E-selectin proteins were detected using an antibody toward the Strep-tag located at the C terminus of each E-selectin protein (Fig. 1*A*), then analyzed by flow cytometry. As illustrated in Fig. 2*A*, E-S6-IgG, E-S6, E-S2, and E-S0 were all capable of binding the KG1a cells to varying degrees. As shown in Fig. 2*B*, E-S6-IgG bound to the ligands on 96% ± 0.8 of the live KG1a population, whereas E-S6 and E-S2 bound to significantly fewer (86% ± 2.1 and 76% ± 2.1, respectively; *p* ≤ 0.05; *n* = *3,* compared with E-S6-IgG). Interestingly, we found that the construct containing only the lectin and EGF domains (E-S0) bound the lowest percentage of KG1a cells (43% ± 6.3; *p* ≤ 0.05; *n* = *3,* compared with all other constructs). E-S6-IgG displayed the strongest fluorescence signal, followed by E-S6, E-S2, and E-S0 (Fig. 2*C*). Additionally, samples stained in the presence of EDTA, which removes divalent cations and inhibits the binding of selectins to their ligands, were included as controls to confirm binding specificity (Fig. 2*A*). Furthermore, we used several increasing concentrations of each construct to test if the binding ability of the weaker constructs would improve upon increasing their concentrations in solution. The results confirmed that the binding trend of each construct remained the same and did not improve for the weaker constructs to the degree that is comparable with the stronger-binding constructs (Fig. S3).

Western blot analysis was conducted to determine the ability of the recombinant E-selectin protein constructs to bind common E-selectin ligands. To this end, PSGL-1 (33) and CD44/HCELL (32) were each immunoprecipitated from KG1a whole cell lysates and subsequently subjected to a Western blot analysis to measure their binding to the E-selectin protein constructs. E-S6-IgG exhibited the strongest staining for both CD44 (Fig. 2*D*, *upper panel*) and PSGL-1 (Fig. 2*D*, *lower panel*) compared with the other constructs. E-S6 exhibited visibly stronger staining to both ligands compared with E-S2 or E-S0 (Fig. 2*D*). Furthermore, identical blots were stained with E-selectin constructs in the presence of EDTA to chelate out the calcium that is essential for binding and binding was abrogated (Fig. S4). Overall, these results suggest that the strongest ligand binding is to the dimeric form of E-selectin and that the SCR domains contribute to this interaction. Because the binding of the E-selectin proteins to PSGL-1 and CD44/HCELL was similar, we chose to focus on the latter for further characterization of the E-selectin proteins.

**Figure 3.**
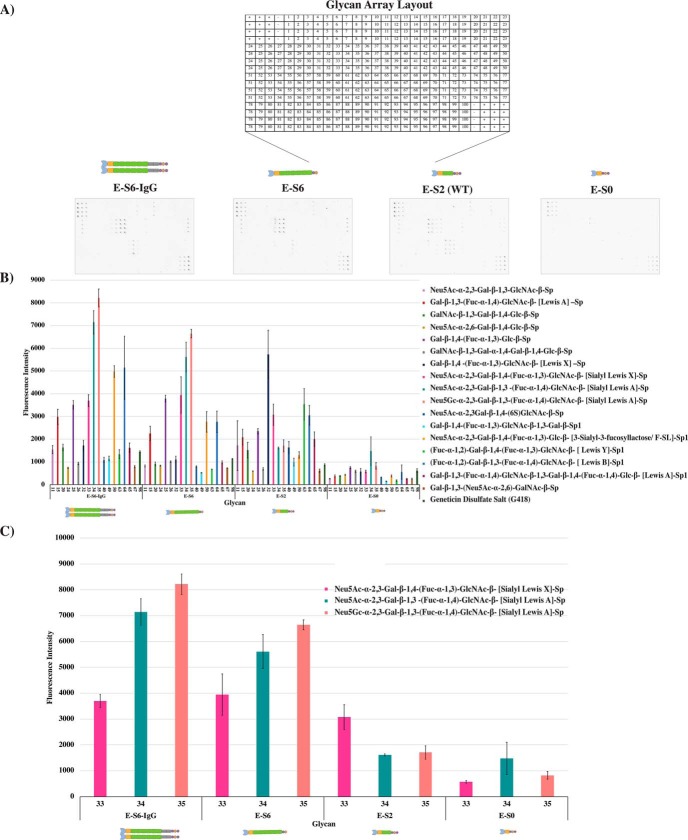
**Glycan microarray analysis.** *A*, *upper panel*, E-selectins (E-S6-IgG, E-S6, E-S2 (WT), and E-S0) were introduced to 4× glycan subarray slides, and each bound construct was detected with biotin anti-His followed by Cy3 Equivalent Dye-Streptavidin. The layout of each array is shown with the location of each glycan, positive (+) and negative (−) controls. *B*, fluorescent intensity of each glycan was determined from quadruplicate samples, and a bar chart was generated and the means are depicted as mean ± S.E.M. for each construct. Each glycan is represented as a number and a color on the *x*-axis. *C*, a subset of the fluorescent intensity highlighting the differences in binding of each E-selectin to Neu5Ac-α-2,3-Gal-β-1,4-(Fuc-α-1,3)-GlcNAc-β- (sialyl-Lewis X)-Sp (33: *magenta*), Neu5Ac-α-2,3-Gal-β-1,3-(Fuc-α-1,4)-GlcNAc-β-(sialyl-Lewis A)-Sp (34: *green*), and Neu5Gc-α-2,3-Gal-β-1,3-(Fuc-α-1,4)-GlcNAc-β-(sialyl-Lewis A)-Sp (35: *peach*). OCH_2_CH_2_Ch_2_NH_2_ and NH(CH_3_)OCH_2_CH_2_NH_2_ linkers are represented as Sp and Sp1, respectively.

Furthermore, glycan-binding microarray analysis was conducted for each E-selectin construct (Fig. 3*A*). An overall decrease was observed in the binding of E-selectin to different glycans upon reducing or omitting the SCR domains (Fig. 3*B*). In particular, shorter E-selectins (E-S2 and E-S0) showed a gradual decrease in the binding to sLe^x^ (Neu5Ac-α-2,3-Gal-β-1,4-(Fuc-α-1,3)-GlcNAc-β) and to two sLe^a^ structures ((Neu5Ac-α-2,3-Gal-β-1,3-(Fuc-α-1,4)-GlcNAc-β-(sialyl-Lewis A) and Neu5Gc-α-2,3-Gal-β-1,3-(Fuc-α-1,4)-GlcNAc-β)) compared with E-S6-IgG and E-S6 (Fig. 3*C*). This indicates that the recognition level of each E-selectin construct toward sLe^x/a^ structures is similar to that toward intact ligands. A list of all the glycans within a microarray is shown in Fig. S5.

**Figure 4.**
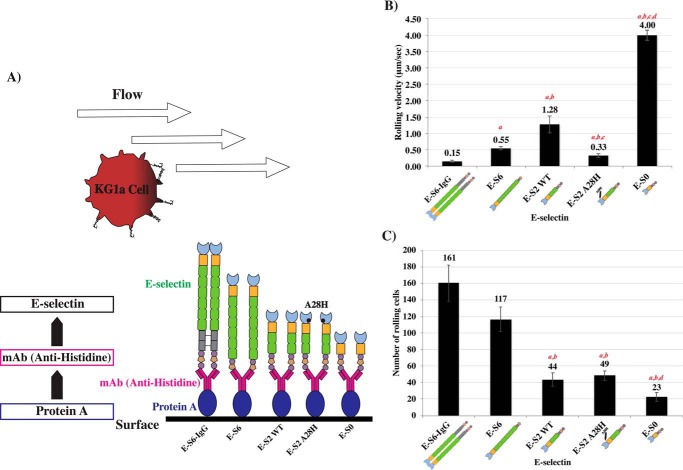
**Cell rolling analysis of KG1a cells on immobilized E-selectin proteins.** *A*, schematic representation of the experimental steps involved in the microfluidics-based flow assay. To ensure the uniform immobilization of each of the proteins, identical concentrations of protein A were deposited on each channel of an uncoated μ slide VI^0.1^ microscopy chamber. This was followed by the addition of anti-histidine antibody and, subsequently, E-selectin proteins were introduced and captured through their C-terminal histidine tags (refer to Fig. S6: each E-selectin can be pulled down equivalently using anti-histidine). KG1a cells in perfusion buffer containing 0.5 mm Ca^2+^ were drawn into the E-selectin–coated channels at various shear stresses (1 dyne/cm^2^, 2 dyne/cm^2^, 3 dyne/cm^2^, 4 dyne/cm^2^, 5 dyne/cm^2^, and 6 dyne/cm^2^) for duration of 30 s each. *B*, adhesion bar graph of rolling velocities (μm/s) from *n* = *3* independent experiments, evaluating the rolling of KG1a on histidine-immobilized E-selectins in the presence of 0.5 mm Ca^2+^, represented as mean ± S.E.M. *C*, bar graph representing the number of KG1a cells rolling on each construct as an alternative measurement for adhesive strength from *n* = *3* independent experiments, represented as mean ± S.E.M. (*n* = *3*; *a* indicates significance compared with E-S6-IgG, *b* indicates significance compared with E-S6, *c* indicates significance compared with E-S2, and *d* indicates significance compared with E-S2-A28H, *p* ≤ 0.05).

We next characterized the differences in binding among the E-selectin protein constructs using a physiological flow-based binding assay. A modified form of the parallel-plate flow-based binding assay (34–42) was used to achieve a quantitative comparison of the binding and rolling velocities of KG1a cells on various immobilized E-selectin protein constructs. In these experiments, E-selectin constructs were deposited at similar concentrations in each channel of a six-channel microfluidic chamber. To position the E-selectin proteins in the correct orientation, we used the histidine tag located at the C terminus of each E-selectin construct (His) (Fig. 1*A*). Each channel was coated with equal amounts of protein A, followed by an antibody against the histidine tag (Fig. 4*A*), which was found to consistently immunoprecipitate each recombinant E-selectin (Fig. S6). The rolling velocities (Fig. 4*B*) and the numbers of rolling cells (Fig. 4*C*) were determined for each E-selectin protein and found to be consistent with our observations above. An analysis of the rolling velocities revealed that E-S6-IgG, followed by E-S6, supported the slowest rolling velocities with 0.15 ± 0.03 μm/s and 0.55 ± 0.05 μm/s, respectively, whereas the shorter proteins supported much faster rolling velocities (1.28 ± 0.26 μm/s for E-S2 and 4 ± 0.16 μm/s for E-S0) (Fig. 4*B*). Longer constructs with six SCR domains supported the highest number of rolling KG1a cells with 161 ± 22 rolling cells on E-S6-IgG and 117 ± 15 on E-S6; significantly fewer cells rolled on the truncated constructs (44 ± 8 cells for E-S2 and 23 ± 5.4 cells for E-S0; *p* ≤ 0.05; *n* = *3*) (Fig. 4*C*). Moreover, similar results were obtained when Fab fragments of the anti-histidine antibody were used in place of the intact antibody (Fig. S7). Interestingly, forced dimerization, induced by using the intact antibody, effectively reduced the rolling velocities for the shorter constructs compared with the Fab fragments, further supporting the influence that dimerization of E-selectin has in promoting binding to its ligands.

Previous studies of the interaction between selectins and their ligands improved our understanding of the mechanical behavior of selectin-ligand interactions (22, 43–55). A major discovery from these studies was the existence of a higher affinity forms of P-selectin (22) and E-selectin (55), which were achieved by extending the conformation of the lectin and EGF domains. To advance our understanding of the domain contributions to the binding behavior of E-selectin, we asked if the weak binding affinity of E-S2 could be enhanced by an equivalent mutation in the lectin domain (A28H) (54) that mimics the force-free extended form of E-selectin (55). The mutated E-S2 (E-S2-A28H) was tested against the other constructs in the same set of assays described above. In the flow cytometric analysis, the number of KG1a bound to E-S2-A28H increased by ∼1-fold (84% ± 4.2) compared with E-S2 and similar to the number of cells bound by E-S6 (86% ± 2.1) (Fig. 2*B*). Additionally, the fluorescence signal of E-S2-A28H (1689 ± 352.5) was only ∼2-fold lower than that of E-S6, whereas the signal of E-S2 was ∼3-fold lower than that of E-S6 (Fig. 2*C*), making the difference between the fluorescence signals of E-S2-A28H and E-S6 insignificant compared with the significant difference observed between E-S2 and E-S6 (*p* ≤ 0.05; *n* = *3*). Moreover, flow-based experiments evaluating the rolling velocities of KG1a cells on immobilized E-S2-A28H showed a significant reduction in the rolling velocity (0.33 ± 0.06 μm/s) (*p* ≤ 0.05; *n* = *3*) compared with E-S2 (rolling velocity = 1.28 ± 0.26 μm/s), and even approaches that of E-S6 (Fig. 4*B*). Together, these data illustrate the importance of the SCR domains, conformational extension, and dimerization in E-selectin binding behavior and in mediating slower rolling velocities.

### Binding kinetics reveal SCR domains, extension, and protein dimerization contribute to the association rate

**Figure 5.**
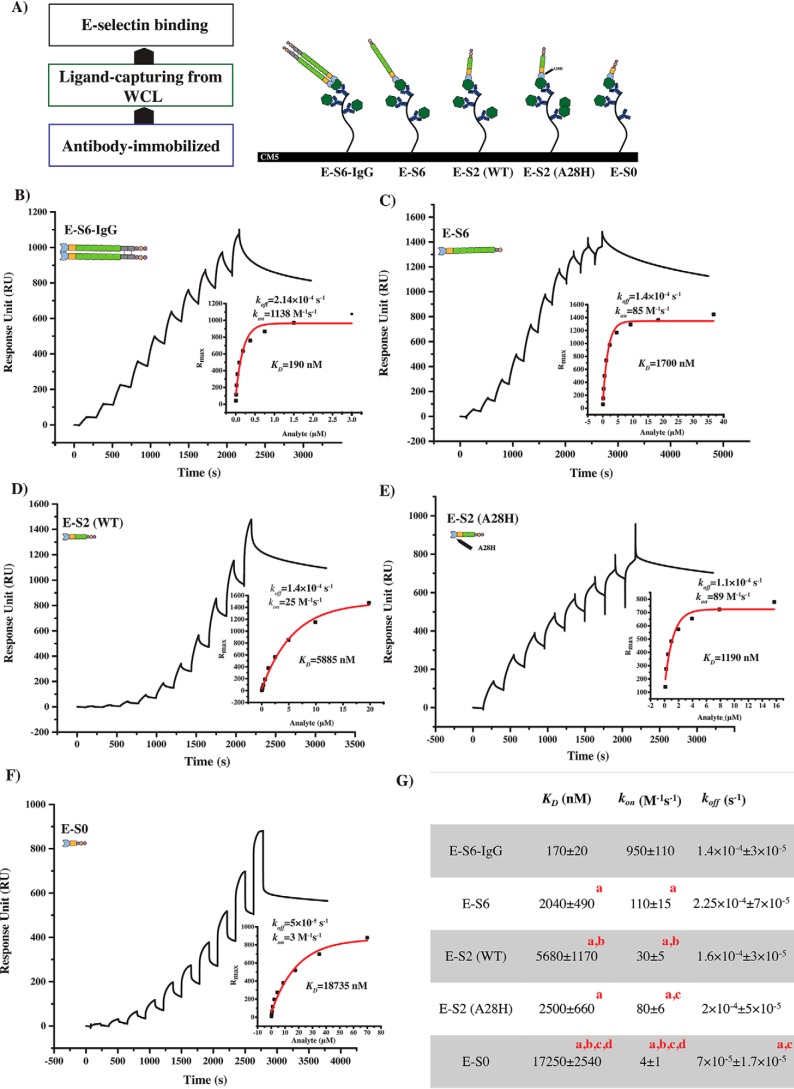
**Rate constants for E-selectin protein binding to CD44/HCELL.** *A*, experimental schematic diagram of the SPR-based real-time binding assay. Step 1, mAb immobilization on CM5 chip; step 2, KG1a lysate injection to capture CD44/HCELL; step 3, injection of various E-selectin proteins. *B*, binding of titrated concentrations of E-S6-IgG to captured CD44/HCELL from KG1a lysates via immobilized Hermes-3 (4113 RU); sensorgram representation of sequential injections of E-S6-IgG at 0.005, 0.01, 0.02, 0.05, 0.09, 0.19, 0.38, 0.75, 1.5, and 3 μm at a flow rate of 20 μl/min for 90 s each, spaced by a 60-s washing step. The lysate injection is not shown. The sensorgram profile was corrected for nonspecific interaction by subtracting isotype control (4466 RU). *K_D_* and *k*_off_ values were determined, as described previously (30). *k*_on_ was calculated using *K_D_* and *k*_off_ values. *C*, binding of E-S6 (monomer form of the full-length E-selectin) to CD44/HCELL injected at different concentrations, 0.1, 0.19, 0.38, 0.77, 1.53, 3.06, 6.12, 12.25, 24.49, and 48.98 μm, using similar experimental conditions to those described in (*B*). The RU for Hermes-3 and its isotype control were 8670 and 6770, respectively. *D*, binding of truncated E-S2 to CD44/HCELL injected consecutively at concentrations 0.04, 0.08, 0.15, 0.31, 0.62, 1.24, 2.48, 4.95, 9.9, and 19.8 μm using similar experimental conditions to (*B*). The RU for Hermes-3 and its isotype control were 8450 and 5090, respectively. *E*, binding of titrated concentrations of E-S2-A28H to captured CD44/HCELL at different concentrations, 0.2, 0.3, 0.7, 1.3, 2.6, 5.2, 10.5, and 20.9 μm, using similar experimental conditions to (*B*). The RU for Hermes-3 and its isotype control were 6680 and 6360, respectively. *F*, binding of the SCR-deficient E-S0 to CD44/HCELL injected consecutively at concentrations of 0.14, 0.27, 0.56, 1.09, 2.19, 4.38, 8.75, 17.50, 35, and 70 μm using similar experimental conditions to (*B*). The RU for Hermes-3 and its isotype control were 6200 and 4900, respectively. *G*, table summarizing the binding constants of the E-selectin proteins to CD44/HCELL from *n* = *3* independent experiments for each E-selectin (*B–F*), reported as mean ± S.E.M. (*n* = *3*, *a* indicates significance compared with E-S6-IgG, *b* indicates significance compared with E-S6, *c* indicates significance compared with E-S2, and *d* indicates significance compared with E-S2-A28H, *p* ≤ 0.05).

We used our previously described real-time surface plasmon resonance (SPR) binding assay (30) to quantitatively evaluate the interaction of the recombinant E-selectin proteins with the native endogenous selectin ligands expressed on the KG1a cells. Specifically, we captured CD44/HCELL from a KG1a cell lysate via the surface-immobilized monoclonal antibodies (mAb) against CD44 (9) (Fig. 5*A*). Then, the E-selectin proteins were injected in sequential concentrations at similar flow rates to determine the equilibrium dissociation binding constant (*K_D_*) and to estimate the association (*k*_on_) and dissociation rate constants (*k*_off_). The captured CD44 bound various forms of recombinant E-selectin constructs, with a *K_D_* of 170 ± 20 nm for the full-length dimeric E-S6-IgG and significantly higher *K_D_* of 2040 ± 490 nm, 5680 ± 1170 nm, and 17250 ± 2540 nm for E-S6, E-S2, and E-S0, respectively (comparing the *K_D_* of E-S6-Ig to other constructs, *p* ≤ 0.05; *n* = *3*) (Fig. 5, *B–D* and *F–G*). To better understand the reason for these differences among the *K_D_* values of each construct, we determined the *k*_on_ and *k*_off_. At the dissociation phase, all of the E-selectin proteins tested showed similar *k*_off_ values, demonstrating that neither dimerization nor the SCR domains are required for the stable binding of E-selectin with CD44/HCELL. Strikingly, in contrast to the dissociation rate, the association rate constants revealed different binding kinetics. Full-length E-S6-IgG exhibited a *k*_on_ (950 ± 110 m^−1^s^−1^) that is ∼9-fold higher (*p* ≤ 0.05; *n* = *3*) than that of monomeric full-length E-S6 (110 ± 15 m^−1^s^−1^). Moreover, shorter constructs displayed significantly lower *k*_on_ values; E-S2 exhibited *k*_on_ values ∼4-fold lower than E-S6 (30 ± 5 m^−1^s^−1^), and E-S0 exhibited *k*_on_ values ∼28-fold lower than E-S6 (4 ± 1 m^−1^s^−1^) (Fig. 5*G*). Furthermore, we confirmed that the binding of the shortest construct (*i.e.* E-S0) at high concentration does not seem to be a result of aggregation (Fig. S8).

The *K_D_* for the binding of E-S2-A28H (Fig. 5, *E* and *G*) to immobilized CD44/HCELL was 2500 ± 660 nm. This *K_D_* is ∼2-fold higher than E-S2 (*K_D_* = 5680 ± 1170 nm). On the other hand, the difference between the affinities of E-S2-A28H and E-S6 (*K_D_* = 2040 ± 490 nm) decreased by only ∼1-fold compared with the ∼3-fold difference between E-S2 and E-S6. The *k*_on_ for E-S2-A28H was ∼3-fold higher (80 ± 6 m^−1^s^−1^) than the *k*_on_ for E-S2 (30 ± 5 m^−1^s^−1^) and approached that of E-S6 (*k*_on_ = 110 ± 15 m^−1^s^−1^) (*p* ≤ 0.05; *n* = *3*) (Fig. 5*G*). Interestingly, the *k*_off_ was not influenced by the mutation in E-S2-A28H (Fig. 5, *E* and *G*). These findings indicate that binding can be improved by extending the lectin and EGF domains, even under conditions where the binding via the SCR domain is weakened. They also suggest that the extended structure contributes to the on rate of E-selectin binding, similar to the role of dimerization and the SCR domains. Consistent with the physiologically relevant flow data above, the SPR results further support that the SCR domains are indeed intrinsically important to the improved binding affinity of E-selectin. They also reveal the critical roles of the SCR domains, protein extension, and oligomerization in influencing the on rate, but not the off rate, of E-selectin binding to its ligands.

## Discussion

Employing a novel silkworm expression system, we were able to purify five different E-selectin proteins in high yield and high purity. We showed that the binding activities of E-selectin to its ligands were improved by increasing the number of SCR domains, by forming an extended lectin and EGF domain structure, and by dimerizing/oligomerizing the protein. These observations were consistently verified by the analysis of these constructs in a number of static and flow-based assays. Our findings further unveiled that all three of these factors contribute to the on rate of E-selectin binding to its ligands, but do not significantly influence its off rate.

The contribution of the SCR domains to ligand binding has been shown previously for P-selectin (56). In that study, similar numbers of neutrophils attached to P-selectin proteins expressing five, six, and nine SCR domains under flow conditions. However, higher concentrations of P-selectin proteins harboring four SCR domains were required for the neutrophils to attach and roll, whereas P-selectin constructs with three or fewer SCR domains failed to bind the neutrophils, even at higher protein densities. Our results from E-selectin are consistent with these findings and established that the SCR domains contribute to the association rate more than to the dissociation rate.

To begin to understand how the SCR domains influence the binding affinity of an E-selectin, it is interesting to note that quite a low conservation of SCR domains exists (only 35%) among the selectins (55), which could potentially have an effect on binding functionality of each selectin toward its ligands. Atomic force microscopy studies revealed that a higher degree of stiffness is associated with the number of SCRs within each selectin implying that these domains act as springs and therefore, L-selectin exhibits the highest level of stiffness (with two SCRs), followed by E-selectin (with six SCRs), and then P-selectin (with nine SCRs) (57). Moreover, the rigidity within the SCR domains is partially provided by the presence of two disulfide bonds on the terminal regions and one central disulfide bond within each SCR domain (11, 55).

Structurally, SCR domains are indeed important for sufficiently extending the lectin domain of E-selectin to access the ligands on cells traveling through the blood. A simple model proposes that the full-length E-selectin tends to be in closer proximity to cells in the blood flow than the truncated constructs, leading to a higher frequency of interactions with their ligands. Additionally, our observations suggest that SCR domains may play a more direct functional role in improving the binding affinity of E-selectin; when E-S6-IgG, E-S6, E-S2, and E-S0 were introduced in solution to their ligands, they each exhibited distinctive binding characteristics. It is likely that the lectin domain is more involved in the actual binding to its sLe^x^-expressing ligand, whereas the SCR domains contribute more to increase the association rate by interacting with other regions of the ligand. It is also possible that the SCR domains might influence the conformation of the selectin to better expose the lectin and EGF domains to facilitate binding to its ligand.

Testing the binding activity of our constructs toward different glycan structures revealed that all the constructs recognize sLe^x^ and sLe^a^ units (Fig. 3), albeit to varying degrees. This is consistent with the data presented in Figs. 2 and 4, suggesting that binding to E-selectin (and thus sLe^x/a^) varies depending on the dimerization and the number of SCR domains. These findings are more likely to support the role of SCR domains in presenting the lectin and EGF domains in the proper conformation and, therefore, supporting the binding functionality of E-selectin. However, further detailed structural studies are needed to confirm this hypothesis.

The crystal structures of P- and E-selectin containing the lectin and EGF domains exhibited two conformational states: bent (22, 47, 55) and extended (22, 55). In the absence of a ligand, the selectin crystals adopted a bent conformation. When sLe^x^ was introduced by soaking it into the bent conformer crystals, it bound to both selectins without any observed changes to the conformation. However, co-crystals formed following the binding of a sulfated fragment of PSGL-1 with P-selectin, or the binding of a glycomimetic of sLe^x^ with E-selectin, resulting in an extended conformation of both selectins (22, 55, 58). This conformational shift of the E-selectin molecule toward a higher affinity extended conformer upon binding to a glycomimetic of sLe^x^ (58) was not observed previously in crystal structures of either selectin in the presence of soaked sLe^x^ (22, 47). Instead of introducing sLe^x^ to bent conformer crystals (22), the binding of a mimetic form of sLe^x^ to E-selectin was performed in a solution, and the resulting complexed molecules were then co-crystallized (55). The extended conformation of the E-selectin/sLe^x^ complex was further confirmed by the observation of a shift in the small-angle X-ray scattering curve, which corresponded to a change in the angle between the lectin and EGF domains.

A mutation in the lectin domain of P-selectin at position A28H was reported to open a split in the lectin domain that triggered a stable version of a higher affinity extended form of the selectin in a force-free fashion (54). Because we observed the functional influence of SCR domains on the binding activity of E-selectin, we tried to improve the binding of the E-selectin molecule bearing only two SCR domains (E-S2) by introducing a similar A28H mutation. The mutated form of this E-S2 (E-S2-A28H) showed improvement in the binding affinity compared with E-S2 and was capable of overcoming the loss of four SCRs by reaching an affinity near that of the full-length monomeric E-selectin molecule (E-S6) with comparable *K_D_* values. Furthermore, this observation is supported by the slower rolling velocity of the KG1a cells on E-S2-A28H in rolling assays compared with the cells rolling on E-S2. These results demonstrate that the extended structure of the lectin and EGF domains is another contributor to selectin binding, even under conditions where the contribution of the SCR domain is compromised. Interestingly, the extended structure acted in a similar manner to the SCR domains in that it specifically enhanced the on rate of E-selectin binding. Although the extended conformer decreases the *K_D_* to similar -fold in P- and E-selectin, the contribution it plays for the association and dissociation rates appears to be different. The extended conformer in P-selectin decreases the dissociation rate (54, 59), whereas in E-selectin, it only influenced the association rate (current study). These results suggest that the extended conformer may play different roles in the binding kinetics between E- and P-selectin.

Species-specific differences in binding strength indicate that mouse E-selectin possesses stronger binding activity than human E-selectin and that this is correlated to the size of the interdomain angle, which is greater in the mouse than in the human form (60). Consistent with these findings, we reported previously that monomeric human E-selectin binds CD44/HCELL transiently with fast on and off rates (30) whereas the monomeric mouse E-selectin, in the current study, bound CD44/HCELL with slow on and off rates and as tightly as the dimeric form.

A number of studies have focused on the impact of selectin dimerization on binding (61–65). Monomeric E-selectin binds similarly to both the dimeric and monomeric forms of PSGL-1, and dimeric E-selectin binds more strongly to dimeric PSGL-1 (66). Similarly, cells expressing PSGL-1 formed much more stable rolling with dimeric P-selectin compared with monomeric P-selectin (67), suggesting that dimeric versions of P-selectin (and PSGL-1) could support the tethering and rolling stability of cells by increasing their chance of forming a new bond after dissociation, *i.e.* “rebinding,” as the cell is tethering, thus prolonging the lifetime of the bond (23). Furthermore, kinetic studies comparing soluble monomeric P-selectin to the membrane (most dimeric) P-selectin revealed fast on/fast off binding for monomeric P-selectin and a biphasic on with a slow off rate for membrane P-selectin (68) which is consistent with several binding analyses of the binding kinetics of dimeric and monomeric P-selectin (69, 70). In our current study, both the dimer and monomer bind with slow on and off rates, whereas the dimer primarily contributes to a significantly slower association rate of binding. These findings also support the important role of E-selectin clustering in its interaction with its ligands during physiological cell adhesion processes.

Overall, our results consistently showed that mouse E-selectin binds with slow on and off rates to native ligands. This is in contrast to the fast on and off rate reported for the human monomeric form of P-, L-, and E-selectin (22, 30, 54, 59, 68, 69, 71, 72). It further showed that SCR domains, conformational extension, and dimerization all collectively contribute to the association rate, whereas the lectin and EGF domains are responsible for the dissociation rate. Under physiological conditions of sheer force, the reported differences in the association rate have dramatic effects on the functional binding behavior of E-selectin. Therefore, it is critical to quantitatively characterize the association and dissociation rates of selectin binding to their well-characterized native ligands. We believe that the silkworm expression system will introduce new capabilities to the selectin field at large to help move it toward more quantitative and structural approaches to understanding these interactions.

## Experimental procedures

### Construction of E-selectins expression vectors

A E-S6-IgG chimera common construct, containing a full-length extracellular region of mouse E-selectin and an Fc region of human IgG1 attached with His_6_ tag, was designed based on the structure of commercially available materials (575-ES, R&D Systems), except for a single mutation that removed the free cysteine residue (S103C; IgG sequence) to eliminate crosslinking. Then, the construct was synthesized and obtained at the institutional core facility, Bioscience Core Lab (KAUST BCL). The C-terminal His_6_ tag (73) was replaced with an Avi double-His-Strep II (73, 74) composite tag. Additionally, the monomer E-selectin (E-S6) was synthesized and obtained similarly to E-S6-IgG (described above), with the addition of a TEV cleavage site, and His_8_ (73) and Strep II tags (74), instead of the double-His-Strep II introduced to E-S6-IgG. E-S6-IgG was also used as the starting template for synthesizing the truncated constructs, E-S2 and E-S0. For the construction of E-S2, the first PCR reaction was completed using the forward primer caccATGAATGCCTCGCGCTTTCTCTCTGC, the overlapping reverse primers CCATCATGCAAAGCTtccggcctgaacgac, tccggcctgaacgacatcttcgaggctcagaaaatcgaatggcacgaacatcatcacc, and aggttaCTCGAGtcaatgatgatggtgatgatgtt for the addition of an Avi tag, a single histidine tag (His_6_), and an XhoI restriction site. Similarly, E-S0 was produced in a PCR reaction identical to the E-S2 PCR synthesis, with the replacement of the reverse primer CCATCATGCAAAGCTtccggcctgaacgac with CCCAACTGTGAGCAAtccggcctgaacgacatcttcgaggctc. Subsequently, the E-S2 and E-S0 constructs were subjected to a second PCR reaction to add Strep-tag, followed by a second polyhistidine, (His_6_), with the forward primer ATGAATGCCTCGCGCTTTCTCTCT and the overlapping reverse primers CCAAGCTCTTGAatgatgatggtgatgatgttcgt, catTCAAGAGCTTGGCGTCATCCGCAGTTCGGTGG, tcaGTGATGGTGATGGTGATGACCACCGAACTGCGga, and aggttaCTCGAGtcaGTGATGGTGATGGTGATGAC. The TEV and Avi tags were included for future work and are not utilized in the current study.

Truncated E-selectin constructs were cloned into a pENTR11 (Invitrogen) vector using the Gateway^TM^ cloning system for generating recombinant expression vectors. Briefly, E-selectin amplicons were digested with XhoI (C∧TCGAG), whereas the pENTR11 (Invitrogen) vector was digested with XhoI and XmnI (GAAnn∧nnTTC) (New England Biolabs). The fragments were directionally cloned into pENTR11 by ligation using T4 DNA ligase (Invitrogen). E-S2-A28H was created by subjecting E-S2-pENTR11 recombinant vector to site-directed mutagenesis. A PCR reaction was performed using the forward primer TACACACATCTGGTGcacATTCAGAACAAGGAA and reverse primer TTCCTTGTTCTGAATgtgCACCAGATGTGTGTA. The Gateway^TM^ LR recombination reaction was performed using LR clonase to produce recombinant pDEST8 expression vectors (Fig. S9) containing the different forms of E-selectins under the transcriptional regulation of a P_PH_ Polyhedrin promoter. Design of all constructs and primers was performed using ApE-A Plasmid Editor.

### Generation of recombinant BmNPVs and purification of recombinant E-selectins

Recombinant pDEST8 expression vectors, constructed as described above, were used to generate recombinant viral DNAs using a BmNPV/T3 bacmid system, as described previously (75). The viral DNAs were isolated using the FlexiPrep kit (Amersham Biosciences), then transfected into the NIAS-Bm-oyanagi2 (BmO2, kindly provided by Dr. Imanishi) cell line using the FuGENE HD transfection kit (Promega, Madison, WI). Then, 4 days after transfection, the culture medium was gathered as recombinant P1 viruses. High-titer virus (P3) stocks were prepared by serial infection following the protocols recommended in the manufacturer's manual (Invitrogen).

The recombinant E-selectins/BmNPVs were infected into the hemocoel of 2-day-old fifth instar silkworm larvae (NBRP, provided by the Institute of Genetic Resources, Kyushu University Graduate School) using a microliter syringe with a 30-gauge needle (Hamilton Co.). At 4 days post infection, the sera from the silkworm larvae were collected in a 15-ml tube containing 20 mm 1-phenyl-2-thiourea. Following brief centrifugation, they were stored at −80 °C until use.

Purification of the recombinant E-selectins was modified from our previously published protocol (76). Briefly, a two-step purification was performed using standard protocols based on the tandem, terminal His and Strep tags. First, the serum diluted with buffer A (20 mm Tris-HCl, pH 7.4, 0.5 m NaCl, 1 mm PMSF) was purified by nickel affinity chromatography with a HisTrap Excel column (GE Healthcare) and eluted by increasing the concentrations of imidazole solution buffers (100–500 mm imidazole). Then, the eluted solution was concentrated (Amicon Ultra-15 3 or 30K filters, Millipore) and diluted with the binding buffer B (100 mm Tris-HCl, pH 8.4, 150 mm NaCl, 1 mm EDTA, PIC (1 tablet/50 ml)) (Roche). A final 50 ml diluted solution was applied to the StrepTrap HP column (GE Healthcare), followed by elution with buffer B, which contained 2.5 mm desthiobiotin. Finally, the purified recombinant E-selectins were dialyzed against 1× PBS (pH 7.4), separated through 10% SDS-PAGE electrophoresis and quantified by Pierce BCA Protein Assay kit.

### Cell lines

Human acute myelogenous leukemia (KG1a) cell line was used as the model for the CD34^+^ HSPCs (ATCC). The cells were cultured in RPMI 1640 media (Gibco) supplemented with 10% fetal bovine serum (FBS) (Cellgro) and 100 units/ml of HyClone penicillin/streptomycin (Invitrogen). These cell lines were grown and maintained in a suspension culture at a density of 1 × 10^6^ cells/ml, under humid conditions in a 37 °C incubator supplemented with 5% CO_2_.

### Flow cytometry

The KG1a cells (at a concentration of 1 × 10^6^ cells/ml) were stained with 10 μg/ml of each of the E-selectins, E-S6-IgG, E-S6, E-S2, E-S2-A28H, or E-S0, in 100 μl HBSS with 5% FBS, in the presence of either 2 mm CaCl_2_ or 20 mm EDTA, at 4 °C for 20 min. Next, the stained cell samples were washed with the same HBSS buffer (containing either 2 mm CaCl_2_ or 20 mm EDTA) one time, and further stained with 10 μg/ml of either Strep-tag II mAb (EMD Millipore), mouse IgG1 PE conjugated isotype control antibody (R&D Systems), or PE rat anti-mouse IgG1 (Clone A85–1) (BD Biosciences) as the secondary control, and then were incubated at 4 °C for 20 min. The samples were then washed two times with the same washing buffer and incubated with 10 μg/ml of PE rat anti-mouse IgG1 (Clone A85–1) for another 20 min at 4 °C. Finally, the stained cells were again washed two times and analyzed using BD FACSCanto (BD Biosciences) and FlowJo software (version 7.6.1).

### Cell lysis and immunoprecipitation (IP)

The KG1a cells were lysed using the membrane disruption method with Triton X-100–based lysis buffer (150 mm NaCl, 50 mm Tris-HCl, pH 7.4) (Fisher Scientific), 1× of protease inhibitor (Roche), 1 mm of PMSF (Sigma Aldrich) and 1% of Triton X-100 (Thermo Fisher Scientific) at a density of 1 × 10^6^ cells/16 μl of lysis buffer, and were rotationally mixed for 1 h at 4 °C. The soluble fraction was collected by centrifugation at 16,000 relative centrifugal force for 30 min at 4 °C. PSGL-1 and CD44 were immunoprecipitated separately from the heterogeneous mixture of proteins by incubation with 50 μl of prewashed Protein G Dynabeads (Thermo Fisher Scientific) complexed with either 3 μg of mouse anti-human CD162 (clone KPL-1) (BioLegend) or 3 μg of mouse anti-human CD44 (clone 515) (BD Biosciences) and mixed rotationally overnight at 4 °C. Next, the supernatants were removed, and the beads were washed three times with lysis buffer chilled to 4 °C.

### Western blotting and native polyacrylamide agarose gel electrophoresis

Protein G Dynabeads with immunoprecipitated PSGL-1 and CD44 were prepared for PAGE by resuspending the beads in 2× NuPAGE lithium dodecyl sulfate sample buffer (Invitrogen) in PBS (Gibco) and 10% β-mercaptoethanol (Sigma-Aldrich). The sample was then heated at 95 °C for 5 min to assess the denaturation process by releasing the capturing antibody with the immunoprecipitated PSGL-1 or CD44 from the protein G beads and into the solution. For the Western blot analysis, the prepared samples were loaded into a 4–20% SDS-polyacrylamide gradient gel (Bio-Rad) in 1× Tris-Glycine SDS buffer (Sigma) as lysates of 1 × 10^7^ KG1a cells/well and transferred to an immunoblot PVDF membrane (Bio-Rad) in 1× Tris-Glycine buffer (Sigma). The resulting membrane was blocked with 5% of nonfat milk in 1× TBST buffer (Cell Signaling Technology) containing 137 mm sodium chloride, 20 mm Tris, and 0.1% Tween 20 at pH 7.6. The prepared blots were stained individually with 1 μg/ml of E-S6-IgG (either homemade from silkworm or produced from NS0 cells) (R&D Systems), E-S6, E-S2, E-S2-A28H, or E-S0 in 1× TBST buffers containing 2 mm CaCl_2_ or 20 mm EDTA and were subsequently stained again with 1 μg/ml Strep-tag II mAb, followed by incubation with 0.057 μg/ml of HRP-conjugated goat anti-mouse IgG (Thermo Scientific) or, for staining the dimeric E-S6-IgG proteins from both silkworm and mammalian cells, directly with HRP-goat anti-human IgG (SouthernBiotech) at 1:10,000 (all staining and washing steps were done in 1× TBST buffers containing 2 mm CaCl_2_ or 20 mm EDTA). Lastly, the membranes were exposed to SuperSignal West Pico Chemiluminescent Substrate (Thermo Scientific) for visualization via the chemiluminescence reactions and imaged using ImageQuant LAS 4000 (GE Healthcare).

To characterize the possible aggregation properties of our E-selectins, protein samples were also prepared for native gel electrophoresis at a final volume of 10 μl in 1× of 4× native PAGE sample buffer (Invitrogen). The prepared samples were loaded into a 10% 10 wells TBE gel (Novex), and electrophoresis was conducted in 1× Tris-glycine buffer for 3 h at 200 voltage. Subsequently, the resulting gel was stained using G-250 Coomassie Brilliant Blue stain (Millipore), then destained in water.

### Glycan array analysis

Sandwich-based protocol was followed in accordance with the manufacturer's instructions using a 4-array glycan chip (GA-Glycan-100–4; RayBiotech). First, each array was blocked using 400 μl of sample diluent at room temperature for 30 min. Next, the sample diluent was removed and a 135 μg/ml solution of each construct (E-S6-IgG, E-S6, E-S2, and E-S0) was prepared in sample diluent containing 2 mm CaCl_2_. 400 μl of each construct was then added to one glycan array. Samples were incubated for 3 h at 4 °C with continuous mixing (low shaking speed ∼60 revolutions per minute (rpm); Thermo Fisher). Each array was washed four times with 800 μl of 1× wash buffer I and two times with 800 μl of 1× wash buffer II; each washing step was performed for 5 min at low shaking speed (∼60 rpm). Next, each array was incubated overnight (4 °C) with 400 μl of biotin anti-His_6_ tag antibody (BioLegend) (prepared in diluent buffer at a final concentration of 1 μg/ml) with low shaking speed (∼60 rpm); the slide was covered with adhesive film to protect it from environment and contamination. The following day, the antibody solution was removed, and each array was washed, as stated above. Next, 400 μl of Cy3 equivalent dye-steptavidin (prepared as 1× in sample diluent buffer) was added to each array and incubated for 1 h at room temperature at low shaking speed (∼60 rpm). Cy3 equivalent dye-steptavidin was then removed, and each array was washed as described above. Subsequently, the gasket defining each array as a separate well was removed, and the slide was soaked consecutively in the following solutions in a slide washer/dryer (RayBiotech): 30 ml 1× wash buffer I, 30 ml 1× wash buffer II, and 30–40 ml milliQ; each wash was performed for 5 min at room temperature. Finally, the residual solution was removed from the surface of the chip and the chip was imaged using the Typhoon Trio variable mode imager (GE Healthcare). Finally, the resulted intensities were measured for each spot using ImageJ software.

### Surface plasmon resonance

Affinity and kinetics assessments of the E-selectins' binding were performed using the Biacore T-100 system on a carboxymethylated (CM5) dextran sensor chip (GE Healthcare) at 25 °C, as described previously (30). First, the system and the flow cells of the sensor chip were washed in two priming steps using filtered (through 0.2 μm filter) and degassed 1× HBS-EP buffer (0.01 m HEPES, pH 7.4, 0.15 m NaCl, 3 mm EDTA, 0.005% v/v surfactant P20) (GE Healthcare). Mouse anti-human CD44 Hermes-3 (Abgent) was immobilized on each flow cell of the CM5 surface using an amine-coupling procedure. In this method, each flow cell was activated by injecting a 1:1 ratio of *N*-hydroxysuccinimide (0.1 m) and 1-ethyl-3-(3-dimethylaminopropyl) carbodiimide hydrochloride (0.4 m) for 8 min at a flow rate of 5 μl/min. Then, 20 μg/ml of the antibody was injected in 10 mm sodium acetate phase (pH 5.0) at a 10 μl/min flow rate, to allow amine-mediated immobilization of the antibody on the CM5 flow cell surface; the resulting amount of immobilized antibody was monitored as indicated in Fig. 5 (4000–9000 RU). Finally, deactivation was achieved by injecting 1 m ethanolamine hydrochloride at a flow rate of 5 μl/min over a total period of 8 min. Using the same procedure, mouse IgG2a κ isotype control (BioLegend) was immobilized on a control flow cell. To prepare for CD44 IP, KG1a cells were lysed at a density of 3 × 10^7^ cells in 300 μl of a lysis buffer containing 1% Triton X-100, 250 mm NaCl, 50 mm Tris base (pH 8.0), and 1 mm CaCl_2_ mixed on a rotator for 1 h at 4 °C. Subsequently, the clear phase was collected by centrifugation at 16,000 relative centrifugal force for 30 min. The system was primed twice using a filtered and degassed running buffer (50 mm Tris, pH 8.0, 1% Triton X-100, 50 mm NaCl, and 1 mm CaCl_2_) to exchange the buffer on the flowing cells. CD44 real-time IP was initiated by injecting the lysate at a 20 μl/min flowrate for 13 min, followed by a 15-min washing step. The E-selectins were titrated in running buffer and injected at a sequence of concentrations, as indicated in Fig. 5. Each injection lasted for 1.5 min at a flow rate of 20 μl/min, and 1-min washing steps were included between the injections. The data analysis was conducted using Biacore evaluation software, as described previously (30), and the kinetics and affinity profiles were generated and analyzed using OriginLab software.

### Cell rolling assay

The cell rolling assay permitted us to evaluate the incorporated shear stress conditions on the physiological interactions of the E-selectins with their ligands. In this assay, the six channels of an uncoated μ-slide VI^0.1^ microscopy chamber (ibidi) were coated with protein A (Thermo Fisher) at 10 μg/ml in HBSS (Gibco) for 16 h. Then each channel was washed in one step. After the channels were completely dry, mouse anti-histidine (AbD Serotec) was added as a second coating at a concentration of 10 μg/ml in HBSS for 1 h at 4 °C, then each channel was washed as described above. The E-selectins (E-S6-IgG, E-S6, E-S2, E-S2-A28H, and E-S0) were added to the channels at 5 μg/ml (diluted in HBSS) and incubated at 4 °C for 16 h to uniformly immobilize them by utilizing the histidine tag located at the C terminus of each E-selectin (Fig. 1*A*). Next, the channels of the chamber were washed and blocked using HBSS containing 1% BSA for 1 h at 4 °C, then washed again. The prepared chamber was placed on the stage of an inverted microscope (Olympus) connected to a CCD camera to visualize and record the rolling events using Cell Sens software. Shear stress within a rectangular channel was calculated as described previously (77) using Equation 1:

(Eq. 1) $$\tau =6\mu Q/a^{2}b$$

where τ is the shear stress (dyne/cm^2^), μ is the viscosity of the fluid (0.00076 Pa.s), Q is flow volume (ml/s), *a* and *b* are the height and width of the channel (0.1 mm and 1 mm), respectively.

The inlet of each channel was connected by a tube (0.8 mm silicone tubing from ibidi) to a cell-containing buffer (1.2 × 10^6^ cells in 1 ml of HBSS containing 1% BSA and 0.5 mm CaCl_2_ or 5 mm EDTA), whereas the outlet was connected to a highly programmable Harvard syringe pump. The suspended KG1a cells were gradually drawn through the channel first at 90 dyne/cm^2^ for 1 min, and then allowed to attach to the immobilized constructs by decreasing the shear stress rate to 0.3 dyne/cm^2^ for 5 min. Next, various shear stress rates were introduced sequentially, corresponding to a sequence of 1 dyne/cm^2^, 2 dyne/cm^2^, 3 dyne/cm^2^, 4 dyne/cm^2^, 5 dyne/cm^2^, and 6 dyne/cm^2^ for 30 s each. The recorded rolling incidents of the KG1a cells on the immobilized E-selectins were analyzed using Imaris software to evaluate the number of rolling cells and their rolling velocities on each E-selectin.

### Statistical analysis

Data are reported as the means ± S.E.M. Statistically significant differences between the means of each E-selectin were resolved using an unpaired Student's *t* test. Statistical significance was defined as *p* ≤ 0.05.

## Author contributions

F. A. A., K. S., and J. S. M. conceptualization; F. A. A., K. S., J. M. L., D. B. A., B. A., S. N., M. T., and J. S. M. data curation; F. A. A., K. S., J. M. L., D. B. A., B. A., S. M. H., S. H., T. K., and J. S. M. formal analysis; F. A. A., K. S., J. M. L., D. B. A., and J. S. M. investigation; F. A. A., K. S., J. M. L., D. B. A., B. A., and J. S. M. methodology; F. A. A., K. S., J. M. L., D. B. A., and J. S. M. writing-original draft; F. A. A. and J. S. M. writing-review and editing; J. M. L., T. K., and J. S. M. resources; T. K. and J. S. M. project administration; S. M. H., S. H., T. K., and J. S. M. supervision; S. M. H., S. H., and J. S. M. validation; T. K. and J. S. M. funding acquisition.

## Supplementary Material

Supporting Information

## Footnotes

2 The abbreviations used are:

HSPC: hematopoietic stem/progenitor cells
sLe^x^: sialyl-Lewis X
EGF: epidermal growth factor domain
SCR: short consensus repeats
SPR: surface plasmon resonance
TEV: tobacco etch virus
HBSS: Hanks' Balanced Salt Solution
TBST: Tris-buffered saline with Tween 20
IP: immunoprecipitation
rpm: revolutions per minute
RU: response units
*K_D_*: equilibrium dissociation binding constant
*k_off_*: dissociation rate constant
*k_on_*: association rate constant.
