# Supplementary material for: Functional binding of E-selectin to its ligands is enhanced by structural features beyond its lectin domain
Source: J Biol Chem. 2020 Jan 16;295(11):3719–33. doi: 10.1074/jbc.RA119.010910 (PMC7076219; doi:10.1074/jbc.RA119.010910)
Supplement: Supporting Information [file supp_295_11_3719__index.html]

Functional Binding of E-selectin to its Ligands is Enhanced by Structural Features Beyond its Lectin Domain — Structural features enhance E-selectin binding — Functional binding of E-selectin to its ligands is enhanced by structural features beyond its lectin domain — Structural features enhance E-selectin binding — Supporting Information 

# Functional binding of E-selectin to its ligands is enhanced by structural features beyond its lectin domain

## Supporting Information

- Supporting Information (to be published online) - Supplemental Methods and Figures
