# Supplementary material for: Functional binding of E-selectin to its ligands is enhanced by structural features beyond its lectin domain
Source: J Biol Chem. 2020 Jan 16;295(11):3719–33. doi: 10.1074/jbc.RA119.010910 (PMC7076219; doi:10.1074/jbc.RA119.010910)
Supplement: Supporting Information [file supp_RA119.010910_155599_2_supp_459441_q47cjz.docx]

**SUPPLEMENTAL INFORMATION FOR THE MANUSCRIPT**

Functional E-selectin Binding is Enhanced by Structural Features Beyond the Lectin Domain

Fajr A. Aleisa^1^, Kosuke Sakashita^1^, Jae Man Lee^2^, Dina B. AbuSamra^1^, Bader Alwan^1^, Shuho Nozue^1^, Muhammad Tehseen^1^, Samir M. Hamdan^1^, Satoshi Habuchi^1^, Takahiro Kusakabe^2^ and Jasmeen S. Merzaban^1,*^

**INVENTORY OF SUPPLEMENTAL INFORMATION**

**SUPPLEMENTAL METHODS**

**Anti-histidine-based immunoprecipitation (IP) of E-selectin constructs**

**Fab fragment preparation of anti-histidine antibody**

**SUPPLEMENTAL FIGURES**

**Supplemental Figure 1- Supplemental Figure 9**

**SUPPLEMENTAL METHODS**

***Anti-histidine-based immunoprecipitation (IP) of E-selectin constructs***

An amount of 50 μL of prewashed protein G dyna beads (Thermo Fisher Scientific) was mixed with 3 μg of mouse anti-histidine (AbD Serotec) were used to immunoprecipitate 5 μg/mL of each recombinant E-selectin. The mixture was incubated overnight at 4 °C with rotational mixing. Next, the supernatants were removed and the beads were treated with 2X NuPAGE lithium dodecyl sulfate sample buffer (Invitrogen) in PBS (Gibco) and 10% *β*-mercaptoethanol (Sigma Aldrich). The sample was then heated at 95 °C for 5 minutes to assess the denaturation process by releasing the capturing antibody with the immunoprecipitated E-selectins. The eluted samples were subjected to subsequent Western blot analysis (see Supplemental Fig. 6).

***Fab fragment preparation of anti-histidine antibody***

Fab fragments of Mouse anti-histidine antibody were obtained using Pierce Mouse IgG_1_ Fab and F(ab’)_2_ Preparation Kit (Thermo Scientific Product No. 44980). An amount of 0.5 mg of the antibody was incubated in digestion buffer containing 3.9 mg cysteine·HCl in 10 mL of the Mouse IgG_1_ Digestion Buffer. Next, fragmented antibody was purified by using Nab^TM^ Protein A Plus Spin Column. Concentration of Fab fragment was estimated by measuring absorbance at 280nm and extinction coefficient of 1.4 was used for the calculation (0.076 mg/mL). Purified samples were analyzed against intact antibody in a 4–20% SDS-polyacrylamide gradient gel (Bio-Rad) in 1X Tris-Glycine SDS buffer (Sigma) (see Supplemental Fig. 7*A*).

**SUPPLEMENTAL FIGURES**

**Supplemental Figure 1: *N*-linked glycosylation in insect and mammalian cells.** *N*-glycan is transferred to an Asparagine residue (Asn) of a nascent protein. (*A*) in insect cells, the structure is processed through a simple pathway to produce paucimannose product. (*B*) in mammalian cells, several glycosyltransferase enzymes are involved in a process to produce more complex glycosylation profiles such as that are further sialylated (red box). Further branching and elongation can take place to produce other types of complex *N*-glycans.

**Supplemental Figure 2: Western blot analysis comparing the binding functionalities of E-S6-IgG from silkworm and mammalian E-S6-IgG (expressed in NS0 cells).** CD44 and PSGL-1, two E-selectin ligands, were immunoprecipitated from KG1a whole cell lysates and subjected to Western blot analysis in order to directly compare staining of E-S6-IgG from silkworm (*A*) and mammalian NS0 cells (*B*). Anti-human IgG conjugated to HRP was used to detect E-selectin proteins bound to E-selectin ligands in the presence of either calcium or EDTA control (data not shown). E-S6-IgG from both expression hosts were similarly capable of specifically staining both E-selectin ligands.

**Supplemental Figure 3: Flow cytometric analysis comparing the concentration dependence in the binding of the various E-selectin proteins to KG1a cells.** (*A*) E-S6-IgG, E-S6, E-S2, E-S2-A28H and E-S0 were tested for their ability to bind to ligands on KG1a cells. 10 μg/mL, 20 μg/mL, 30 μg/mL, 40 μg/mL or 50 μg/mL of each construct was used to stain KG1a cells. Mouse monoclonal anti-strep antibody (followed by a fluorescently labeled antibody against anti-strep) was used to detect the E-selectin protein bound to ligands on the surface of KG1a cells in the presence of calcium. The percentages of KG1a cells bound to each of the E-selectin proteins (*B*) and the geometric means of the fluorescence signals (*C*) were determined from *n=3* experiments.

**Supplemental Figure 4: Western blot analysis of E-selectin protein binding to immunoprecipitated PSGL-1 and CD44 in the presence of EDTA.** KG1a lysates were prepared and PSGL-1 and CD44 were immunoprecipitated (IP) and subjected to a Western blot analysis. The resulting blots (*upper panel*: CD44; *lower panel*: PSGL-1) were stained with 1μg/mL of E-S6-IgG, E-S6, E-S2 or E-S0, as indicated in the figure, in the presence of calcium (Fig. 2*D*) or EDTA (shown here); blots stained by EDTA showed no binding activity, confirming the binding specificity. Anti-strep mAb was used to detect bound E-selectin for subsequent chemiluminescence detection using HRP-conjugated anti-mouse IgG.

**Supplemental Figure 5:** Glycan microarray layout in numerical order and the corresponding glycan in each location. This array was used for data presented in Fig. 3 of the main manuscript file.

**Supplemental Figure 6: Western blot analysis confirming the ability of anti-histidine to immunoprecipitate each recombinant E-selectin.** E-S6-IgG, E-S6, E-S2, E-S2*-*A28H and E-S0 were immunoprecipitated at identical concentrations using anti-histidine antibody (*n=2*). (*A*) Immunoprecipitated samples were subjected to Western blot analysis; their molecular weights are indicated in the figure with red arrows. Heavy (50 kDa) and light (25 kDa) chains corresponding to denatured anti-histidine antibody are detected in each lane. (*B*) Blots of the flow-through from the immunoprecipitation were simultaneously analyzed and no proteins in the flow-through fractions were detected, confirming the efficiency of anti-histidine in capturing each recombinant protein.

**Supplemental Figure 7: Cell rolling analysis comparing immobilization of the E-selectin constructs using intact anti-histidine antibodies to Fab fragments of the anti-histidine antibody.** (*A*) Analytical SDS-PAGE of fragmentation procedure of mouse anti-histidine (anti-his) antibody (AbD Serotec) showing intact mouse anti-his antibody and Fab fragment obtained from anti-his antibody after digestion and purification using Pierce Mouse IgG1 Fab Preparation kit. Similar to the scheme outlined in **Figure 4A**, both intact and Fab fragments were immobilized using Protein A. Intact (blue bars) or Fab fragments (orange bars) of the mouse anti-his antibody were then used to immobilize E-S6-IgG, E-S6 and E-S2 E-selectin constructs. KG1a cells were then introduced and allowed to roll over the immobilized constructs under flow of 1 dyne/cm^2^, 2 dyne/cm^2^, 3 dyne/cm^2^, 4 dyne/cm^2^, 5 dyne/cm^2^ and 6 dyne/cm^2^ shear stresses for a duration of 30 seconds each. The rolling velocities (*B*) and number of rolling cells (*C*) were recorded and plotted. Data were obtained from *n=3* independent experiments.

**Supplemental Figure 8: E-S0 protein binding specificity to immobilized CD44/HCELL.** Binding of a high concentration of E-S0 (17.68 μM) to captured CD44/HCELL from KG1a lysates via immobilized CD44 antibody (Hermes-3 clone; 4059 RU) on a CM5 chip was evaluated by SPR. The *k_off_* of binding was calculated to be 1.41×10^-4^ s^-1^. Injections of KG1a lysate and E-S0 in the presence of calcium (1 mM) or EDTA (10 mM) were performed at a flow rate of 10 μL/min for 380 seconds, spaced by washing step. Injection start and end of KG1a lysate, E-S0 in the presence of EDTA and calcium are indicated by arrow heads in the presented sensorgram. The sensorgram profile was corrected for non-specific interaction by subtracting the isotype control sample (6444 RU).

**Supplemental Figure 9: Genetic map of recombinant pDEST8 vector used for expression of E-selectin proteins.** pDEST8 destination vector for insect cell expression with an internal *ccd*B toxin gene for negative selection. An ampicillin resistance gene is used for selection. *att*R1 and *att*R2 recombination site cassettes were utilized to perform an LR gateway reaction (Gateway™ cloning technology) for inserting the desired E-selectin construct from an entry (pENTR11) vector (e.g., E-S6) to obtain a recombinant pDEST8 expression vector with the E-selectin construct of interest under the transcriptional control of a polyhedrin (P_PH_) promoter. Circular pDEST8 plasmid map and linear E-S6 map were drawn by ApE plasmid software and further modified by Adobe Illustrator.
